# A Structure-Based Approach for Mapping Adverse Drug Reactions to the Perturbation of Underlying Biological Pathways

**DOI:** 10.1371/journal.pone.0012063

**Published:** 2010-08-23

**Authors:** Izhar Wallach, Navdeep Jaitly, Ryan Lilien

**Affiliations:** 1 Department of Computer Science, University of Toronto, Toronto, Ontario, Canada; 2 Banting and Best Department of Medical Research, University of Toronto, Toronto, Ontario, Canada; 3 Donnelly Centre for Cellular and Biomolecular Research, University of Toronto, Toronto, Ontario, Canada; Dr. Margarete Fischer-Bosch Institute of Clinical Pharmacology, Germany

## Abstract

Adverse drug reactions (ADR), also known as side-effects, are complex undesired physiologic phenomena observed secondary to the administration of pharmaceuticals. Several phenomena underlie the emergence of each ADR; however, a dominant factor is the drug's ability to modulate one or more biological pathways. Understanding the biological processes behind the occurrence of ADRs would lead to the development of safer and more effective drugs. At present, no method exists to discover these ADR-pathway associations. In this paper we introduce a computational framework for identifying a subset of these associations based on the assumption that drugs capable of modulating the same pathway may induce similar ADRs. Our model exploits multiple information resources. First, we utilize a publicly available dataset pairing drugs with their observed ADRs. Second, we identify putative protein targets for each drug using the protein structure database and in-silico virtual docking. Third, we label each protein target with its known involvement in one or more biological pathways. Finally, the relationships among these information sources are mined using multiple stages of logistic-regression while controlling for over-fitting and multiple-hypothesis testing. As proof-of-concept, we examined a dataset of 506 ADRs, 730 drugs, and 830 human protein targets. Our method yielded 185 ADR-pathway associations of which 45 were selected to undergo a manual literature review. We found 32 associations to be supported by the scientific literature.

## Introduction

Adverse drug reactions (ADRs), often informally referred to as side-effects, are rare complex physiologic phenomena that involve various molecular processes [1]. Understanding these processes may greatly impact the fields of drug discovery and personalized medicine through the development of safer drugs, the discovery of new bio-markers, and the identification of new uses for existing drugs. Factors such as the patient's genetic polymorphism, personal history, and environmental exposure as well as drug kinetics, treatment dosage, and molecular metabolism often contribute to ADRs through a direct or indirect perturbation of biological pathways [2]. While some ADRs result from the desired interaction between drugs and their primary targets, in the majority of cases these effects are caused by promiscuous off-target binding of the drug [3].

Several recent studies have investigated the promiscuous relationship between drugs, targets, and observed ADRs (in this manuscript we will use the terms ADR and side-effect interchangeably). Fliri et al. studied the relationships between side-effect profiles of drugs, their chemical structure, and the organism response. They clustered drugs according to their side-effect profiles and clustered side-effects according to the biological systems of their associated drugs (*e.g.*, immune system) thereby linking side-effects and interaction patterns of drugs [4]. In a follow-up study they explored the above relations as a mechanism for predicting sets of side-effects for new drug candidates [5]. Their findings reinforce the hypothesis that structurally similar drugs are likely to induce similar side-effects. Campillos et al. identified alternative targets for known drugs under the hypothesis that structurally similar drugs sharing similar side-effect profiles were also likely to share targets [6]. Keiser et al. analyzed the relationship between protein targets and their ligands using a ligand-based similarity metric that groups together seemingly unrelated proteins [7]. In another study, the same group constructed a model to identify alternative drug labels (or functions) for known drugs by comparing their binding promiscuity [8]. They explored the similarity between drugs and native ligands as an indication for possible binding promiscuity and the use of this information to suggest alternative drug targets. In general, the studies mentioned above utilized ADR profiles as a feature set or fingerprint to predict new drug targets. In each case, the biological process underlying the ADR remained hidden.

Recently, Xie et al. [9] have used virtual docking to study ADRs related to the cholesteryl ester transfer protein (CETP) and identified possible off-target interactions for a set of CETP inhibitors. Their method uses a known 3D protein structure of the primary target to characterize the binding site of the drugs. Then, it identifies potential off-targets by searching for other proteins having similar binding sites. Possible interactions between the resulting set of proteins and the drugs are then studied using virtual docking. In a subsequent work, Durrant et al. [10] augmented that method by adding an evolutionary model to account for protein sequence homology. A substantial limitation of these two approaches is their reliance on the availability of a 3D structure of the primary target. Thus, this method may not be feasible when studying many popular drug targets [11] for which no 3D model yet exists. An approach that does not require any structural knowledge of the primary target was suggested by Yang et al. [12]. In their work, they used virtual docking to propose possible interactions between a set of 845 proteins and a set of 162 drugs all known to induce at least one of four ADRs. Similar to Xie et al., they aimed to identify off-target proteins involved in the appearance of ADRs under the hypothesis that drugs causing the same ADR may target the same proteins. Scheiber et al. [13] analyzed the structural similarity of drugs associated with similar ADRs and identified common chemical sub-structures that may be involved in the induction of ADRs. In a related work from the same group [14], cheminformatics target prediction methods were used to identify potential off-targets for drugs that share the same ADRs. Then, pathways were related to ADRs based on direct mapping between predicted targets and pathways.

In this work, we developed a computational framework for proposing associations between the ADRs of clinically approved drugs and the modulation of underlying biological pathways. In contrast to the work of Xie et al. [9] and Yang et al. [12], our model exploits the assumption that drugs capable of modulating similar pathways may have similar ADR profiles. Under this assumption, an ADR may be associated with a pathway when structurally different drugs, known to induce the same ADR, bind proteins in the same pathway. Using pathway information to connect between ADRs and predicted protein-drug interactions provides two advantages. First, it allows our model to observe cases in which different drugs modulate the same protein target and second, it allows us to identify cases in which the modulation of a pathway via different proteins affects the same biological process. Furthermore, proteins that participate in several pathways can implicate multiple biological processes with a single ADR. We reveal ADR-pathway associations by exploiting three knowledge bases: (i) the SIDER database of ADR profiles for drugs [15], (ii) the KEGG database of biological pathway annotation [16], and (iii) the PDB database of protein structures [17]. Each drug is mapped to several potentially affected pathways by docking the ligand into a set of pathway annotated human proteins.

The comparison of docking profiles for different drugs producing the same ADR allows us to exploit both evidence of binding and non-binding in building a consistent, minimal hypothesis. For example, consider the case where two drugs produce the same ADR. If only one of the two drugs is predicted to interact with pathway X, then it is less likely that pathway X is solely responsible for the ADR. The drug that does not interact with pathway X provides evidence that disrupting the pathway is not necessary (although it may be sufficient). We refer to the information provided by this non-interacting prediction as negative evidence. The proposed interactions between drugs and pathways along with the known co-occurrence of drugs and ADRs are then used to learn associations between pathways and ADRs. Our method demonstrates the ability to draw correlations between ADRs and pathways, despite the virtual docking limitations, and the incomplete catalog of biological processes. Future improvements in virtual docking algorithms and expansion of our knowledge of biological processes should make our method more effective.

## Results

**Figure 1:**
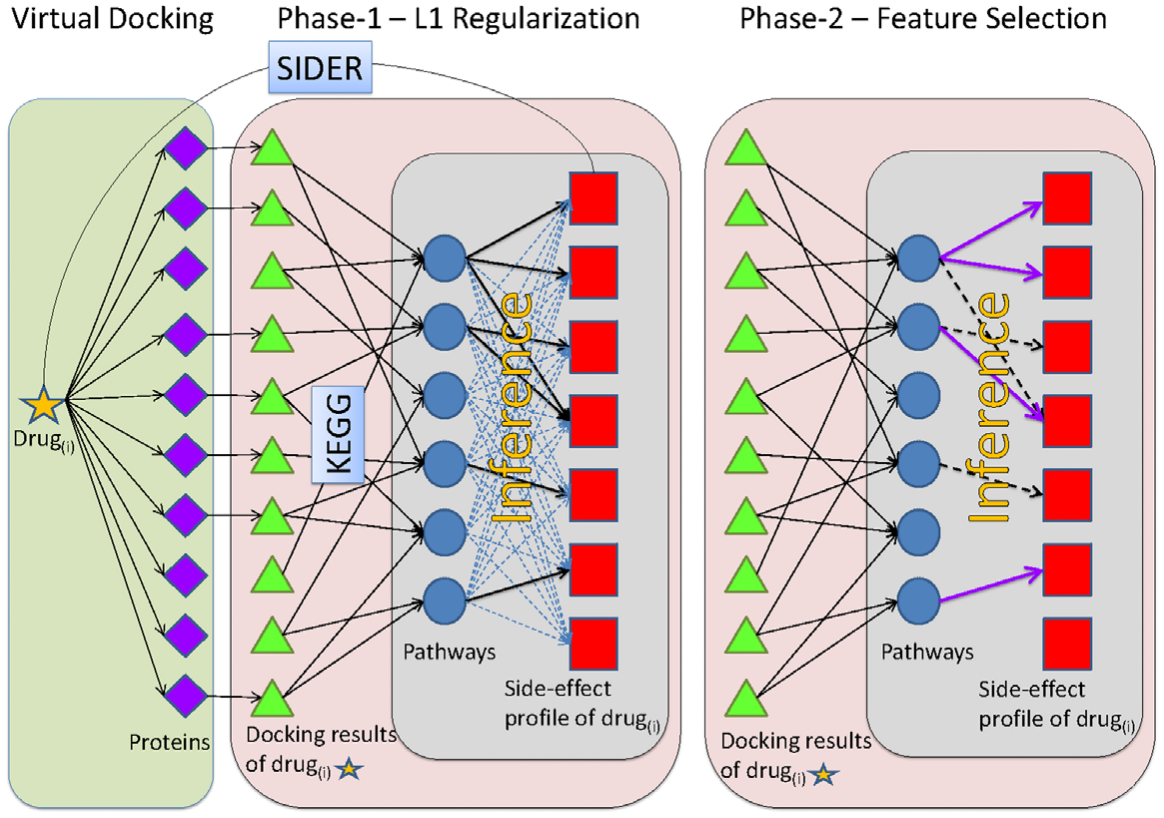
An illustration of the inference method. Drug-pathway interactions are inferred from the results of protein-ligand docking. The KEGG database [16] is used to map proteins to biological pathways. The SIDER database [15] associates drugs with their observed ADRs. In the docking phase each drug (orange star) is docked against each protein (purple diamond) producing a set of docking results (green triangles). Then, two phases of logistic regression are used to select those associations that are statistically significant. In phase-I, logistic regression with L1-regularization is used to infer a set of informative connections between pathways (blue circles) and ADRs (red squares). In phase-II, a second logistic-regression model selects the associations selected from phase-I that are statistically significant under multiple hypothesis correction (see Methods section).

**Figure 2:**
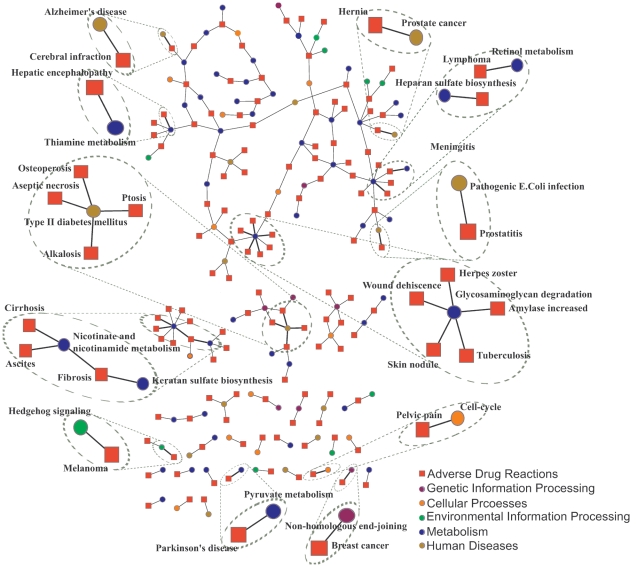
An illustration of the network of pathway-ADR associations inferred by our model. Side-effects are represented as red squares and pathways as blue circles. The full list of 185 associations is available at Table S3. The 22 associations most strongly supported by the literature are circled. Pathways are colored by their KEGG categories.

We developed a computational model that associates ADRs with biological pathways. The model is summarized in this paragraph (full details appear in the Methods section). We used the empirically observed relations between drugs and ADRs, the mapping of target proteins into pathways, and the virtual normalized docking results of drugs onto experimentally determined human protein structures to construct a graphical representation of these relations (Figure 1). The current study utilized 730 small-molecule clinically-approved drugs [15] and 830 target proteins with solved structures [17]. The protein targets were associated with 176 human related pathways extracted from the KEGG database [16]. The drugs were associated with 506 ADRs extracted from the SIDER database [15]. Our computational model learns a set of connections from ADRs to pathways consistent with the observed drug-ADR relations. We note that for some drugs, the ADRs listed in the SIDER database are not traditional ADRs. For example, viral and bacterial infections, such as herpes and tuberculosis, are listed as ADRs but clearly, do not result from direct perturbations of a biological pathway. These ADRs reflect the observations of viral/bacterial infections while patients were under the administration of corresponding drugs. Viral infections may be listed as ADR if a drug perturbs some biological mechanisms and, as a result, increases vulnerability to viral infections. Alternatively, an ADR may not reflect a causative relation. For example, cancer or HIV patients having a stressed immune system, may suffer from increased vulnerability to infections. Consequently, cancer or HIV related drugs may be associated with infectious related ADRs without any causative underlying mechanism. In the results below we demonstrate several such examples. For clarity, we will refer to these biological pathway-ADR associations as simply associations. The learning of these associations employed a 2-phase logistic-regression model with L1-regularization and feature selection [18], [19]. This method was designed to avoid overfitting the small number of input samples by using techniques that heavily penalize complex models. As a result, the model identifies a small informative set of associations for which there is the most evidence. Utilizing a multiple testing correction with a false-discovery rate of 2% our model identified a set of 185 associations worthy of further investigation (Figure 2 and Table S3). These 185 associations involve 90 pathways and 121 ADRs and represent an extremely small fraction of the 89,056 possible pathway-ADR associations (176 pathways and 506 ADRs).

### Analysis of the inferred associations

Validating associations in the predicted set is a challenging task (see the Discussion). In this work, we manually reviewed relevant scientific literature for existent evidence of correctness of our predicted associations. Clearly, the most fundamental limitation of this approach is that we can only support associations already discovered while novel associations suggested by our model will not have any direct support in the literature. In order to facilitate the process of a manual literature review, we first discarded associations for which relevant literature is sparse. In the filtering process, we analyzed the 185 inferred associations using associative text-mining over the biomedical literature. Similar to Fliri et al. [5] we used the frequency at which the association's terms appear in scientific publications as supporting evidence of correctness. Prior to performing each search, the terms of the association were expanded to include equivalent MeSH terms (http://www.nlm.nih.gov/mesh). Then, for every association we performed a PubMed search (http://www.ncbi.nlm.nih.gov/pubmed) for entries containing both terms of the association (*i.e.*, the biological pathway and the ADR). These associations were ranked by the number of hits and the highly ranked associations were chosen to undergo a manual literature review. While this text-mining approach has been used in previous studies [5], [20], the technique does have some limitations. First, it is more likely to return a hit when the two phrases are directly related. Second, the method can only validate previously observed associations; consequently, the inability to validate an association does not imply that it is false, it may simply be unknown (see the Discussion section). Despite these caveats, text mining can provide evidence in support of identified associations. Of the 185 associations identified by our model, 45 exceeded our threshold of having at least 5 PubMed hits and were selected for manual review. After manual examination of the relevant literature, we propose that 22 associations are supported and 10 have slightly less support but remain worthy of further investigation (Table 1). All drug names related to the validated associations appears in Table S9. The full set of relations between drugs, proteins, pathways, and ADRs, is provided in File S1 as a Cytoscape [21] file. We stress that this PubMed-based filtering was only used to facilitate a thorough manual literature review. By selecting a subset of associations that had sufficient annotations in the literature we able to focus on those associations more likely to be valid. Nevertheless, associations not passing the 5-hit threshold may still be correct. For example, the associations of skin nodule and the GAG degradation pathway or aseptic necrosis and the Type-II diabetes pathway did not pass the 5-hit threshold yet were supported by scientific evidences. Another important clarification is that the identification of an association does not, of course, necessarily imply causality. A causal relation may be partial such that the inferred pathway is *involved* in the occurrence of the ADR but is not the sole cause for it. Also, since the ADR data is simply a record of ADR observations coincident with the administration of drugs, non-causative relations may exist in the data and may be found by our model. For example, a pathway may characterize a group of patients for which the ADR is likely to be observed (see the association of hernia with the prostate-cancer pathway below). In the remainder of this section, we discuss some of the associations identified by our method and supported by the scientific literature. For brevity, we list only a limited set of supporting references for each association below. The complete set of references can be found in Table S1 and File S2.

**Table 1:** Associations supported by the literature.

Side-effects	Pathways
Cerebral infarction	Alzheimer's disease
Osteoporosis	Type II diabetes mellitus
Lymphoma	Retinol metabolism
Hernia	Prostate cancer
Parkinson's	Pyruvate metabolism
Breast cancer	Non-homologous end-joining
Pelvic pain	Cell cycle
Fibrosis	Nicotinate and nicotinamide metabolism
Hepatic encephalopathy	Thiamine metabolism
Melanoma	Hedgehog signaling pathway
Prostatitis	Pathogenic Escherichia coli infection
Alkalosis	Type II diabetes mellitus
Stria	Heparan sulfate biosynthesis
Tuberculosis	Glycosaminoglycan degradation
Herpes zoster	Glycosaminoglycan degradation
Cirrhosis	Nicotinate and nicotinamide metabolism
Ascites	Nicotinate and nicotinamide metabolism
Meningitis	Heparan sulfate biosynthesis
Wound dehiscence	Glycosaminoglycan degradation
Amylase increased	Glycosaminoglycan degradation
Fibrosis	Keratan sulfate biosynthesis
Ptosis	Type II diabetes mellitus
Aseptic meningitis	Systemic lupus erythematosus
Lymphoma	Heparan sulfate biosynthesis
Skin carcinoma	Lysosome
Alkalosis	Biosynthesis of unsaturated fatty acids
Hyperparathyroidism	Autoimmune thyroid disease
Fibrosis	Metabolism of xenobiotics by cytochrome P450
Vitamin-D deficiency	Autoimmune thyroid disease
Skin carcinoma	Androgen and estrogen metabolism
Rigmentary retinopathy	Sulfur metabolism
ESR increased	Parkinson's disease

The 32 associations supported by the literature. (Top) The 22 associations with stronger support. (Bottom) The 10 associations with moderate support (see Table S1 for a full reference list).

#### ADRs associated with the glycosaminoglycan degradation pathway

**Figure 3:**
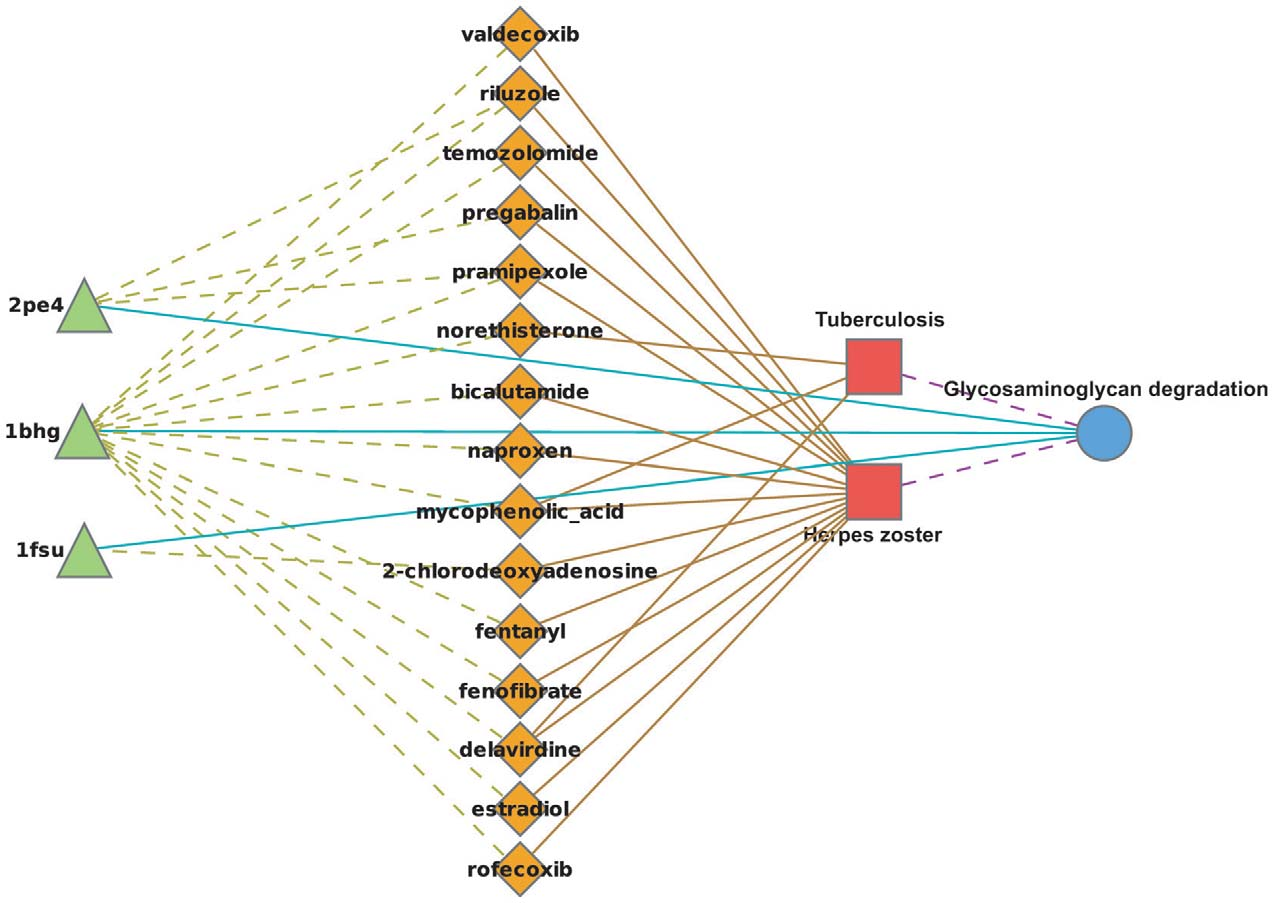
GAG-related ADRs. The illustration represents proteins as green triangles, drugs as orange diamonds, ADRs as red squares, and pathways as blue circles. Protein-ligand interactions as predicted by virtual docking are represented as green dashed lines. Inferred pathway-ADR associations are represented by purple dashed lines. Observed ADR-drug pairs come from the SIDER database and are represented by solid brown lines. Finally, KEGG labels connect proteins to biological pathways and are represented as blue lines.

Glycosaminoclycan (GAG) proteins are abundant in the extracellular matrix and cell membrane and are the first host macromolecules encountered by most infectious agents [22]. Our model associates eight ADRs with the GAG degradation pathway from which six were supported in the literature. These include three ADRs of bacterial or viral infection. Tuberculosis (TB) is a bacterial infection that most commonly affects the respiratory system [23]. GAG proteins have shown to be involved in bacterial and viral infection, including TB, by exploiting GAG proteins to mediate the attachment to target cells [22], [23]. Interestingly, there is also an association between the herpes zoster virus (HZV) and the GAG degradation pathway. Several studied have demonstrated a connection between viral infection, particularly herpes, and GAG proteins [24], [25]. Furthermore, inspection of the suggested interactions between drugs associated with TB and HZV and proteins in the GAG pathway showed that in both TB and HZV infections a single beta-glucuronidase lysosomal enzyme (1BHG) plays a central role (Figure 3). Our model also suggests the association between meningitis infection and the heparan sulfate biosynthesis pathway. Heparan sulfate is a member of the GAG family and is known to be involved in bacterial and viral infection [26]. Thus, for reasons similar to the above, perturbation of the heparan sulfate biosynthesis may increase the risk of meningitis. These three infection related ADRs present an interesting case. Each of the three infection related ADRs is supported by a relatively independent set of drugs. Of the 27 drugs predicted to interact with the GAG degradation pathway, 23 are associated with only one of the three infection related ADRs (Figure S1). This suggests that the three associations were independently inferred.

Other ADRs associated with the GAG degradation pathway are wound dehiscence, amylase increased, and skin nodules. GAG proteins are involved in wound healing and thus may be involved in the occurrence of wound dehiscence [27]. Elevated serum amylase level is one of the indicators of acute pancreatitis [28], an inflammation of the pancreas that has been associated with the GAG pathway [29]. Skin nodules are associated with abnormal level of GAG proteins and particularly heparan sulphate proteoglycan [30], [31].

#### ADRs associated with the nicotinate and nicotinamide metabolism pathway

The nicotinate and nicotinamide (NAD) pathway is involved in the synthesis, utilization and/or degradation of nicotinate and nicotinamide. Our model associates eight ADRs with the NAD pathway from which fibrosis, cirrhosis, and ascites were supported in the literature (Table S1). Interestingly, these three ADRs are clinically related. Fibrosis is the cumulation of excessive collagen in an organ and the formation of scar tissue [32], cirrhosis is an advanced form of liver fibrosis and is characterized by formation of a fibrous scar [33], and ascites is the cumulation of excessive fluid in the abdominal cavity and has been shown to be associated with cirrhosis [34], [35]. Similar to the GAG degradation pathway example, each of the three fibrosis related ADRs were supported by a relatively independent set of drugs (Figure S3).

#### ADRs associated with the type-II diabetes mellitus pathway

Type-II diabetes mellitus (diabetes hereafter) is a disorder of insulin resistance or insulin deficiency characterized by high serum glucose levels [36]. Our model associates four ADRs with the diabetes pathway: osteoporosis, aseptic necrosis, alkalosis, and ptosis, all supported in the literature (Table S1). Particularly interesting are the first two associations. Osteoporosis is a bone disorder characterized by an increased risk of fractures due to a reduction in the bone density [37]. The relation between osteoporosis and diabetes have been established in several studies and particularly, diabetic osteopathy, an increased fracture risk in diabetes patients [38], [39]. Inspecting the Anatomical Therapeutic Chemical classification [40] of the osteoporosis related drugs showed that 8 drugs were classified as corticosteroids, a class of drugs that have been associated with both steroid-induced osteoporosis and diabetes [41]. Aseptic necrosis (AN) is a disease characterized by the death of cells in bones due to lack of blood circulation [42]. While fewer indications of possible connection between diabetes and AN appear in the literature, its relation to osteoporosis provides support to this association. Furthermore, an analysis of the drugs associated with osteoporosis and AN showed that most of them were associated with only one of the ADRs, thereby suggesting a rather independent inference of these two related bone disease (Figure S2).

#### Hernia – Prostate cancer pathway

**Figure 4:**
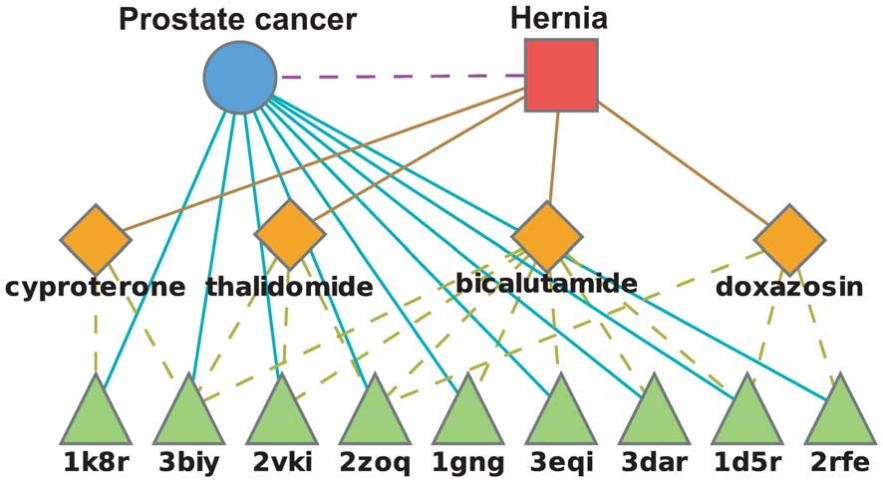
Relations between hernia and the prostate cancer pathway. An illustration of the model's suggested interactions between drugs coincident with hernia and proteins belong to the prostate cancer pathway. This is an example of a *non-causative association* where drugs listing prostate-related disease as their therapeutic indication indeed interact with proteins in the prostate cancer pathway. Since patients suffering from prostate cancer are likely to experience a post-operative hernia, an association between hernia and prostate cancer emerges. Node and edge representation is the same as Figure 3.

A hernia is a protrusion of a tissue or part of an organ through the cavity that normally contains it. The prostate cancer pathway mainly characterizes key molecular alterations in prostate-cancer cells implicating carcinogen defenses, growth-factor-signaling pathways, and androgens [16]. The occurrence of inguinal hernia is a common phenomenon after radical retropubic prostatectomy (the removal of the prostate gland) [43], [44]. While our model suggests a connection between hernia and prostate cancer it is unlikely that perturbations of the prostate related pathways will result in a hernia. This is a demonstration of a *non-causative association*. A plausible explanation for this association is that our data includes prostate-related drugs that list hernia as an ADR. Indeed, there are four such drugs (Figure 4) that all list prostate-related disease as their therapeutic indication [15] and are suggested by virtual docking to interact with prostate cancer related proteins. Since a hernia may occur secondary to surgery, it is likely that the ADR *hernia* was simply reported as an observed phenotype for patients treated with prostate cancer related drugs.

#### Parkinson's disease – Pyruvate metabolism pathway

**Figure 5:**
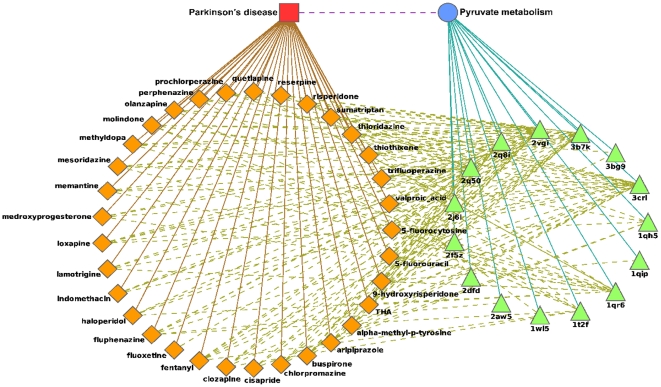
Relations between Parkinson's disease and the pyruvate metabolism pathway. An illustration of the model's suggested interactions between drugs coincident with Parkinson's disease and proteins belonging to the pyruvate metabolism pathway. Node and edge representation is the same as Figure 3.

Parkinson's disease (PD) is a progressive neurological disorder characterized by a large number of motor and non-motor features [45]. Increasing evidence indicates that oxidative stress may play a crucial role in the pathogenesis of PD and that pyruvate deficiency, among other anti-oxidant agents, is significantly involved [46], [47]. Our model suggests 33 drugs associated with PD to interact with 15 proteins from the pyruvate metabolism pathway (Figure 5) out of which 23 drugs are nervous-system agents (Table S2). The promiscuity of these drugs is supported by the fact that 19 of 23 are psychoactive drugs which are well known for their binding promiscuity [47], [48].

#### Melanoma – Hedgehog signaling pathway

Melanoma is a malignant tumor of melanocytes. The Hedgehog signaling pathway plays important roles in determining cell fate, pattern formation, proliferation, and differentiation. Alteration of the pathway has been implicated in a number of human cancers, including melanoma [49], [50].

#### Breast cancer – Non-homologous end-joining pathway

Non-homologous end-joining (NHEJ) is one of the major pathways involved in repairing double-strand breaks (DSB) in DNA. Polymorphisms in NHEJ genes have been shown to be associated with breast cancer [51], [52]. The role of breast cancer related genes, BRCA1 and BRCA2, in the NHEJ pathway suggests that the mechanisms involved in DNA DSB repair are of particularly important during breast tumorigenesis [53].

### Validation tests

The associations suggested by our model were based on its ability to identify meaningful correlations in imperfect virtual docking results. Of course, validated associations could have been discovered randomly, independent of the information provided by the docking results and the structure of the biological network. In order to estimate the accuracy of the docking, to demonstrate that the docking results convey useful information, and to evaluate the likelihood of discovering true associations by chance, we preformed the following sets of experiments.

#### Ranking benchmarks

In order to test how well the docking algorithm ranks active ligands we examined the ranking performance for the 16 DrugBank [54] drug-target pairs that also exist in our dataset. For each ligand, we tested how well the docking algorithm was able to rank drugs when docked into their known targets. In 7 cases, the known interacting drug was ranked higher than one standard deviation from the mean score (z-score greater than one, see Table S8). The theoretical probability of observing such an event (drawing 7 or more numbers greater than 1 from a normal distribution in 16 trials) is less than 0.0016. As a second test, we used the DUD benchmark [55] to asses the ability of the docking algorithm to rank active ligands. DUD is considered the gold standard for benchmarking the ranking provided by virtual docking algorithms. We measure ranking success using the area under the curve (AUC) of the decoys verses actives ranking. Using the DUD benchmark, the docking algorithm achieved a median AUC value of 0.8717. In 15 out of 35 DUD test cases, the AUC was greater than 0.9 (See Figure S4).

#### Randomized control for docking results

We assessed the information content of the virtual docking by performing 1000 randomized trials. In each trial, we randomly shuffled the mapping between the drugs and their docking results (note that this is equivalent to shuffling the mapping between drugs to ADRs) and used this random data as the input for our model. A comparison of the number of returned associations by our predictive model (185) to the distribution of returned associations from the 100 randomized experiments (mean 116.47 and standard deviation 18.45) showed that there was less than 0.01% chance our number of results were drawn from random unstructured data. This implies that there is structure to the virtual screening data.

#### Randomized control for associations

We demonstrated the ability of our model to retrieve associations that are supported by the literature. We evaluated the significance of the identified associations using a randomized control. Ideally, we could randomize the input data, use our algorithm to predict associations, and then assess the correctness of each association via an expert. Performing 100 randomization trials would require the infeasible task of manually evaluating up to 18,500 associations. Instead, we determined if the number of PubMed supported hits in our results (45 out of 185) was statistically different from the expected number of supported associations when drawn at random. We emphasize, that in this experiment we use the PubMed hit count in a different manner than described before. Initially, we used the PubMed hit count to reduce the number of associations which underwent a manual literature review while in this case, we use it as a substitution for the actual manual review. All terms in the random associations were drawn only from those that appeared in the 185 predicted associations (90 pathways and 121 ADRs). As before, for each random association we expanded the search terms using MeSH before querying PubMed. The expected distribution of random associations with at least 5 PubMed hits (mean 37.79, standard-deviation 4.85) suggests a less than 5% chance that our results were drawn from the random data distribution.

## Discussion

The encouraging results presented in this manuscript come despite several limitations. Our method utilizes computational predictions of protein-ligand binding across the human proteome. This requirement means our results are affected by three factors, the availability of protein structures, the accuracy of virtual docking, and the complicating effects of genetic polymorphisms. Although the largest possible set of publicly available human protein structures contains only 830 macromolecules, we were able to identify significant pathway-ADR associations that involve the selected proteins. Virtual docking still struggles with computing true binding energies; however, our model relies on the easier task of separating active binders from decoys. Many docking algorithms, including the eHiTS software utilized in this work [56], are well suited for this ranking task (See Figure S4). Finally, although genetic polymorphisms play an important role in ADRs, we propose that in many cases docking into a wild-type protein and a genetic variant may show grossly similar results. Furthermore, the effects of a genetic variation may be indirect where an ADR emerges secondary to the interaction of a variant protein and a ligand perturbed wild type protein. In this case, our method does not require docking to the genetic variant. The above limitations restrict our list of associations from being complete; but, there are several interesting associations among those identified.

Many problems in machine learning are difficult because they utilize a small number of training samples to fit a large number of features. In our work, it is difficult to identify pathway-ADR associations using the small number of ADRs observed for each drug. In order to adequately deal with a modest set of positive examples, our model utilizes two phases of logistic-regression (Figure 1). In the first phase we use L1-regularization [19] to select an initial set of associations. An L1-penalty term is commonly used in continuous model selection to identify a small set of informative features. L1-regularization reduces the risk of over-fitting by biasing the feature weights towards zero, thereby only allowing associations with strong evidence to have non-zero weights [18]. Regularization of this type is particularly important when the number of possible features exceeds the number of observations. In the second phase, we perform a traditional logistic regression using the features selected in phase one. Using the forward-selection backward-elimination algorithm, the AIC model selection criteria, and a multiple-hypothesis correction we reduce the number of features to a set that remains statistically significant.

Experimentally validating associations between ADRs and pathways is a challenging task since true validation is likely to require in-vivo experiments similar to clinical trails of drug candidates. The predicted associations fall into three classes: true-positive (TP), false-positive (FP), and false-false-positive (FFP). TPs are associations identified by our model, are inherently true, and are known to be true. FPs are associations identified by our model yet are inherently false. FFPs are associations identified by our model and are inherently true, but are as yet unknown to the scientific field and may therefore be misinterpreted as false. While one long range aim of our work is to reveal unknown associations (FFP), by definition it is impossible to validate FFPs without conducting additional experiments. Although our current method is unable to validate FFPs, our use of associative text-mining and manual evaluation allows us to support 32 (of 185) associations as likely TPs.

The aim of this manuscript is to introduce a computational framework for identifying pathway-ADR associations. Our approach is based on predicting the targets of a drug's promiscuous binding using a structural model and then connecting these interactions with biological pathways. Associations are determined using a statistically grounded approach to inference. The initial results presented in this manuscript are promising and we envision that hypotheses generated by our model may guide future research.

## Methods

### Preparation of data sets

#### Drugs

A list of drugs and their corresponding ADRs was obtained from the SIDER database [15]. Structural models of all drugs were retrieved from the PubChem database (http://pubchem.ncbi.nlm.nih.gov) using PubChem's compound ID (CID). If no corresponding structure was found in the database, a 3D model was generated with OpenBabel [57] using the drug's SMILES string [58]. In order to increase the likelihood of successful docking, ligands were filtered by molecular weight and flexibility. We removed drugs which had: (i) a molecular weight outside the range of 100–800 Daltons or (ii) 10 or more rotational bonds. This filtering step yields 730 drugs most compatible with virtual docking (Table S7).

#### Protein Targets

The structures of protein targets satisfying the following criteria were obtained from the Protein Data Bank [17]:

- The structure was solved by either X-ray crystallography or NMR spectroscopy.
- Structures solved by X-ray crystallography have a resolution better than 3Å.
- The protein sequence contains more than 50 amino acids.
- The source organism is human.
- The protein target is an enzyme as indicated by the presence of an Enzyme Commission (EC) number [59].
- The protein has a KEGG annotation [16].

The set of proteins was clustered using the BLASTclust algorithm [60] removing redundant structures sharing more than 90% sequence similarity over 90% of the sequence length. Finally, all selected structures were striped of ligands and salts. This results in set of 830 protein targets (Table S5).

#### Human pathways

We used the KEGG database [16] to build a set of human pathways and their corresponding known protein structures. The 830 protein receptors represent 176 KEGG pathways (Table S4).

#### Side-effects

A list of drugs and their corresponding ADRs was obtained from the SIDER database [15] (version 2009-06-19). In order to deal with similar ADRs that appear under slightly different names in the SIDER database, we first stemmed all ADR phrases (extracting the base part of a word or phrase) using the WordNet lexical database [61]. Then, we measured the Levenshtein distance [62] between all pairs (the minimal number of single character edit operations required to transform one term to the other) and grouped ADRs with an edit distance smaller than two. For a given drug, we removed an ADR if any of the following were satisfied:

- The ADR has a “post-marketing” label (*i.e.*, the ADR was only reported after the drug's approval).
- The frequency of the ADR is less than 1% after subtracting the placebo frequency, if available.
- The ADR is associated with fewer than 3 drugs or is associated with more than 5% (approximately 36) of the drugs.

This procedure yielded a set of 506 ADRs (Table S6). Following Fliri et al. [4] we discard the frequency information of the selected ADRs and regard their occurrence as binary.

### Docking

Automatic docking, while generally less successful than expert guided docking, has recently shown to be viable for a large diverse set of receptors [63]. For every protein target we first identify its two largest pockets using LIGSITE*^csc^* [64]. It has been suggested that in proteins having known binding sites, in 80.9% of the cases the largest pocket is the binding site and in 92.7% of the cases the binding site is one of the two largest ones [65]. Therefore, we dock each ligand into the two largest pockets using the eHiTS docking algorithm [56] (Version 6.2). The docking is performed with full ligand flexibility, examination of all possible protonation states, and a clipping box of 15*Å* around the center of the binding pocket. All other parameters assume their default values. The docking score for each drug-target pair is the better of the two pocket docking attempts. Since docking scores may scale differently when using multiple receptors, we use the z-score to normalize the docking results. Finally, we use the docking results to generate a list of drug-target pairs where for a given pair the drug is expected to bind and influence the protein target. For each drug, we sort the docking results and keep only those proteins where the docking score was better than one standard deviation above the mean. This results in retaining only the top scoring drug-protein pairs for each drug.

### Inference Method

We use logistic regression to study the relations between drug-activated pathways and ADRs (Figure 1). All putative drug-pathway interactions are inferred by protein-ligand docking. The drug-pathway interaction is the sum of docking scores over all proteins belonging to the pathway. These putative interaction scores are then combined with drug-ADR observations to generate candidate pathway-ADR associations.

Statistically significant associations are selected using two phases of logistic regression. In phase one, we perform a logistic regression between the drug-pathway interactions and the drug's observed ADRs. To perform logistic regression, we utilized the L1-regularized logistic regression code of Kim et al. [19]. The regularization parameter, 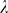, restricts the number of non-zero weights. The likelihood of over-fitting is minimized because only those features having the strongest evidence in the data can have non-zero weights. We use a relative regularization parameter, 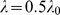, where 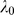 is the smallest regularization value that yields all-zero regression coefficients. In phase two, the pathway-ADR associations having non-zero weights were passed through a second logistic regression to determine their statistical significance. This process included the forward-selection backward-elimination method for variable selection, the AIC model selection criteria [66], and the Benjamini-Hochberg multiple testing correction method [67] with a false discovery rate of 2%. All phase two statistical tests were used as implemented in the R programming environment (http://www.r-project.org).

## Supporting Information

Table S1 The 32 associations supported by the literature. (Top) The 22 associations with stronger support. (Bottom) The 10 associations with moderate support. (References appear in File S2.)
(0.09 MB EPS)

Table S2 Parkinson's related drugs classified as nervous-system agents according to the ATC classifications.
(0.04 MB RTF)

Table S3 The 185 associations inferred by the model.
(0.16 MB RTF)

Table S4 176 pathways used in this work.
(0.09 MB RTF)

Table S5 830 protein structures used in this work.
(0.28 MB RTF)

Table S6 506 ADRs used in this work.
(0.23 MB RTF)

Table S7 730 drugs used in this work.
(0.25 MB RTF)

Table S8 Normalized scores of drug-primary-target pairs in our dataset. Pairs were selected using the DrugBank database [4] (references appear in File S2).
(0.07 MB EPS)

Table S9 The 32 associations supported by the literature with the corresponding drug names. (Top) The 22 associations with stronger support. (Bottom) The 10 associations with moderate support.
(0.06 MB RTF)

Figure S1 Drugs listing tuberculosis, herpes-zoster, and meningitis as ones of their ADRs. The illustration demonstrates the degree of overlap in which drugs coincide with more than one of the three bacterial/viral-related ADRs. The low overlap suggests that inferences of the associations between the three related ADRs and the GAG degradation pathway were highly independent of each other. Node and edge representation is the same as Figure 1.
(0.28 MB EPS)

Figure S2 Drugs listing osteoporosis and aseptic necrosis as ones of their ADRs. The illustration demonstrates the degree of overlap in which drugs coincide with more than one of the three bacterial/viral-related ADRs. The low overlap suggests that inferences of the associations between the three related ADRs and the type-II diabetes mellitus pathway were mostly independent of each other. Node and edge representation is the same as Figure 1.
(6.29 MB EPS)

Figure S3 Drugs listing fibrosis, cirrhosis, and ascites as ones of their ADRs. The illustration demonstrates the degree of overlap in which drugs coincide with more than one of the three bacterial/viral-related ADRs. The low overlap suggests that inferences of the associations between the three related ADRs and the NAD metabolism pathway were highly independent of each other. Node and edge representation is the same as Figure 1.
(5.61 MB EPS)

Figure S4 Ranking benchmark of eHiTS [1] and AutoDock-Vina [2] docking algorithms using the DUD benchmark set [3] (references appear in File S2).
(0.19 MB EPS)

File S1 Cytoscape file.
(0.16 MB GZ)

File S2 Supplementary bibliography.
(0.08 MB RTF)
