# Supplementary material for: A Structure-Based Approach for Mapping Adverse Drug Reactions to the Perturbation of Underlying Biological Pathways
Source: PLoS One. 2010 Aug 23;5(8):e12063. doi: 10.1371/journal.pone.0012063 (PMC2925884; doi:10.1371/journal.pone.0012063)
Supplement: Table S2 — Parkinson's related drugs classified as nervous-system agents according to the ATC classifications. (0.04 MB RTF) [file pone.0012063.s002.rtf]

ATC sub-class (nervous system)	PubChem compound ID (CID)	Drug name	
Anesthetics	3345	fentanyl	
Analgesics	3345	fentanyl	
	5358	sumatriptan	
Antiepileptics	3121	valproic acid	
	3878	lamotrigine	
Psycholeptics	23897	molindone	
	2477	buspirone	
	2726	chlorpromazine	
	2818	clozapine	
	3372	fluphenazine	
	3559	haloperidol	
	3964	loxapine	
	4078	mesoridazine	
	4585	olanzapine	
	4748	perphenazine	
	4917	prochlorperazine	
	5002	quetiapine	
	5073	risperidone	
	5452	thioridazine	
	5454	thiothixene	
	5566	trifluoperazine	
	60795	aripiprazole	
Psychoanaleptics	3386	fluoxetine	
	4054	memantine	
