# Supplementary material for: A Structure-Based Approach for Mapping Adverse Drug Reactions to the Perturbation of Underlying Biological Pathways
Source: PLoS One. 2010 Aug 23;5(8):e12063. doi: 10.1371/journal.pone.0012063 (PMC2925884; doi:10.1371/journal.pone.0012063)
Supplement: Table S3 — The 185 associations inferred by the model. (0.16 MB RTF) [file pone.0012063.s003.rtf]

ADRs	Pathways	
  
Cerebral infarction	Alzheimer’s disease	
Osteoporosis	Type II diabetes mellitus	
Blood dyscrasia	Cytokine-cytokine receptor interaction	
Lymphoma	Retinol metabolism	
Hernia	Prostate cancer	
Tuberculosis	Glycosaminoglycan degradation	
Lymphopenia	Pyrimidine metabolism	
Aseptic meningitis	Systemic lupus erythematosus	
Myocarditis	Steroid biosynthesis	
Lymphoma	Heparan sulfate biosynthesis	
Parkinson’s	Pyruvate metabolism	
Skin carcinoma	Lysosome	
Alkalosis	Biosynthesis of unsaturated fatty acids	
Hyperparathyroidism	Autoimmune thyroid disease	
Cirrhosis	Nicotinate and nicotinamide metabolism	
Blood dyscrasia	Notch signaling pathway	
Breast cancer	Non-homologous end-joining	
Skin carcinoma	Androgen and estrogen metabolism	
Pelvic pain	Cell cycle	
Fibrosis	Keratan sulfate biosynthesis	
Fibrosis	Nicotinate and nicotinamide metabolism	
Pigmentary retinopathy	Sulfur metabolism	
Ptosis	Type II diabetes mellitus	
Hepatic encephalopathy	Thiamine metabolism	
Melanoma	Hedgehog signaling pathway	
Pulmonary infiltration	Sphingolipid metabolism	
Herpes zoster	Glycosaminoglycan degradation	
Periodontitis	DNA replication	
Amylase increased	Glycosaminoglycan degradation	
Ascites	Nicotinate and nicotinamide metabolism	
Fibrosis	Metabolism of xenobiotics by cytochrome P450	
Prostatitis	Pathogenic Escherichia coli infection	
Alkalosis	Type II diabetes mellitus	
Uterine hemorrhage	Parkinson’s disease	
Alkalosis	Caffeine metabolism	
Vitamin d deficiency	Autoimmune thyroid disease	
Esr increased	Parkinson’s disease	
Hypertrichosis	Drug metabolism - other enzymes	
Esr increased	Glycosaminoglycan degradation	
Stria	Heparan sulfate biosynthesis	
Ocular infection	Galactose metabolism	
Skin atrophy	Heparan sulfate biosynthesis	
Meningitis	Heparan sulfate biosynthesis	
Hypophosphatemia	Glycosaminoglycan degradation	
Wound dehiscence	Glycosaminoglycan degradation	
Ocular infection	Ribosome	
Status epilepticus	Lysosome	
Enteritis	Nucleotide excision repair	
Malabsorption	Keratan sulfate biosynthesis	
Pathological fracture	Autoimmune thyroid disease	
Skin nodule	Glycosaminoglycan degradation	
Proctitis	Systemic lupus erythematosus	
Renal tubular acidosis	Thiamine metabolism	
Osteoporosis	Ribosome	
Scleritis	RNA degradation	
Periodontal disease	Vitamin B6 metabolism	
Ischemic colitis	Type I diabetes mellitus	
Peripheral vascular disorder	mTOR signaling pathway	
Vaginal hemorrhage	Parkinson’s disease	
Osteoporosis	Ubiquitin mediated proteolysis	
Malignant hyperthermia	Thiamine metabolism	
Nail disorder	Heparan sulfate biosynthesis	
Cerebral infarction	Phenylalanine, tyrosine and tryptophan biosynthesis	
Retinopathy	Hypertrophic cardiomyopathy (HCM)	
Sinus tachycardia	Glycosaminoglycan degradation	
Allergic rhinitis	Sphingolipid metabolism	
Siadh	Arachidonic acid metabolism	
Fungal dermatitis	Nicotinate and nicotinamide metabolism	
Gum hemorrhage	Heparan sulfate biosynthesis	
Diabetic ketoacidosis	Nicotinate and nicotinamide metabolism	
Hyperpigmentation	Cytokine-cytokine receptor interaction	
Supraventricular tachycardia	N-Glycan biosynthesis	
Blood dyscrasia	Ubiquinone and other terpenoid-quinone biosynthesis	
Aseptic necrosis	Type II diabetes mellitus	
Electrolyte imbalance	Antigen processing and presentation	
Fat embolism	Drug metabolism - other enzymes	
Aseptic necrosis	Ribosome	
Nasal septum perforation	Drug metabolism - other enzymes	
Hypophosphatemia	T cell receptor signaling pathway	
Skin carcinoma	Viral myocarditis	
Renal tubular acidosis	Calcium signaling pathway	
Cervical erosion	B cell receptor signaling pathway	
Renal insufficiency	Maturity onset diabetes of the young	
Gum hemorrhage	Pathogenic Escherichia coli infection	
Papilledema	Drug metabolism - other enzymes	
Papilledema	T cell receptor signaling pathway	
Pulmonary infiltration	Thiamine metabolism	
Serum sickness	Sulfur metabolism	
Benign prostatic hyperplasia	Hematopoietic cell lineage	
Myasthenia gravis	Thiamine metabolism	
Intestinal ulcer	Hedgehog signaling pathway	
Serum sickness	Maturity onset diabetes of the young	
Ards	Citrate cycle (TCA cycle)	
Endocarditis	Hematopoietic cell lineage	
Sinus headache	Hematopoietic cell lineage	
Galactorrhea	Primary immunodeficiency	
Tendinitis	Amyotrophic lateral sclerosis (ALS)	
Myositis	Primary bile acid biosynthesis	
Cholelithiasis	mTOR signaling pathway	
Allergic rhinitis	Amino sugar and nucleotide sugar metabolism	
Myocarditis	Primary bile acid biosynthesis	
Tetany	Nucleotide excision repair	
Megaloblastic anemia	Cell adhesion molecules (CAMs)	
Ards	Pentose phosphate pathway	
Right upper quadrant pain	Hematopoietic cell lineage	
Enterocolitis	Glycolysis / Gluconeogenesis	
Siadh	Huntington’s disease	
Hairy tongue	Maturity onset diabetes of the young	
Hypopigmentation	Methane metabolism	
Esr increased	Taste transduction	
Pseudotumor cerebri	Ribosome	
Hirsutism	Riboflavin metabolism	
Allergic rhinitis	Phenylalanine, tyrosine and tryptophan biosynthesis	
Periodontal abscess	Hypertrophic cardiomyopathy (HCM)	
Fecal impaction	RNA degradation	
Blepharoconjunctivitis	Vitamin B6 metabolism	
Aseptic meningitis	Fatty acid elongation in mitochondria	
Duodenitis	Viral myocarditis	
Cryptococcosis	Keratan sulfate biosynthesis	
Gum hemorrhage	Chondroitin sulfate biosynthesis	
Cholelithiasis	Adipocytokine signaling pathway	
Paralytic ileus	Complement and coagulation cascades	
Fecal impaction	Tyrosine metabolism	
Aphonia	Caffeine metabolism	
Pigmentary retinopathy	Taste transduction	
Fat embolism	Adipocytokine signaling pathway	
Supraventricular tachycardia	Methane metabolism	
Vulvovaginitis	Valine, leucine and isoleucine biosynthesis	
Skin atrophy	Cyanoamino acid metabolism	
Hirsutism	Pentose phosphate pathway	
Hypermagnesemia	Maturity onset diabetes of the young	
Pseudomembranous colitis	Taste transduction	
Hydronephrosis	Amino sugar and nucleotide sugar metabolism	
Macrocytosis	Taste transduction	
Blepharoconjunctivitis	Riboflavin metabolism	
Hirsutism	Fructose and mannose metabolism	
Impetigo	Alanine, aspartate and glutamate metabolism	
Eye redness	Glycosphingolipid biosynthesis - globo series	
Pulmonary infiltration	Pantothenate and CoA biosynthesis	
Tonsillitis	Aminoacyl-tRNA biosynthesis	
Serum sickness	Pantothenate and CoA biosynthesis	
Breast enlargement	Glyoxylate and dicarboxylate metabolism	
Seborrhea	Heparan sulfate biosynthesis	
Folliculitis	Adherens junction	
Benign prostatic hyperplasia	Aminoacyl-tRNA biosynthesis	
Status epilepticus	Fructose and mannose metabolism	
Allergic alveolitis	Nicotinate and nicotinamide metabolism	
Telangiectasia	Cyanoamino acid metabolism	
Ectropion	Keratan sulfate biosynthesis	
Night blindness	Aminoacyl-tRNA biosynthesis	
Scleritis	Cyanoamino acid metabolism	
Duodenitis	Phenylalanine, tyrosine and tryptophan biosynthesis	
Sinus headache	Aminoacyl-tRNA biosynthesis	
Folliculitis	Spliceosome	
Allergic rhinitis	Dorso-ventral axis formation	
Strabismus	Nicotinate and nicotinamide metabolism	
Splenic infarction	Glycosphingolipid biosynthesis - lacto and neolacto series	
Glossodynia	ABC transporters	
Cholelithiasis	Neuroactive ligand-receptor interaction	
Pure red cell aplasia	Cyanoamino acid metabolism	
Pure red cell aplasia	Glycosphingolipid biosynthesis - globo series	
Gum hemorrhage	D-Glutamine and D-glutamate metabolism	
Blepharoconjunctivitis	Other glycan degradation	
Onycholysis	Jak-STAT signaling pathway	
Fat embolism	Glycosphingolipid biosynthesis - globo series	
Proctitis	Pantothenate and CoA biosynthesis	
Hydronephrosis	Pantothenate and CoA biosynthesis	
Hepatomegaly	Glycosphingolipid biosynthesis - globo series	
Miliaria	Heparan sulfate biosynthesis	
Cholelithiasis	Ascorbate and aldarate metabolism	
Sick sinus syndrome	D-Arginine and D-ornithine metabolism	
Periodontitis	Ascorbate and aldarate metabolism	
Onycholysis	alpha-Linolenic acid metabolism	
Lymphopenia	Taurine and hypotaurine metabolism	
Hernia	Ubiquinone and other terpenoid-quinone biosynthesis	
Fecal incontinence	D-Glutamine and D-glutamate metabolism	
Ophthalmoplegia	Ubiquinone and other terpenoid-quinone biosynthesis	
Burning sensation	Ascorbate and aldarate metabolism	
Periodontal disease	RIG-I-like receptor signaling pathway	
Nail disorder	Ubiquinone and other terpenoid-quinone biosynthesis	
Photopsia	Nicotinate and nicotinamide metabolism	
Leukocytoclastic vasculitis	beta-Alanine metabolism	
Lymphopenia	Ubiquinone and other terpenoid-quinone biosynthesis	
Hypertrichosis	Cyanoamino acid metabolism	
Aseptic meningitis	Ubiquinone and other terpenoid-quinone biosynthesis	
