# Supplementary material for: A Structure-Based Approach for Mapping Adverse Drug Reactions to the Perturbation of Underlying Biological Pathways
Source: PLoS One. 2010 Aug 23;5(8):e12063. doi: 10.1371/journal.pone.0012063 (PMC2925884; doi:10.1371/journal.pone.0012063)
Supplement: Table S4 — 176 pathways used in this work. (0.09 MB RTF) [file pone.0012063.s004.rtf]

 Pathways	
ABC transporters	
Acute myeloid leukemia	
Adherens junction	
Adipocytokine signaling pathway	
Alanine, aspartate and glutamate metabolism	
Allograft rejection	
alpha-Linolenic acid metabolism	
Alzheimer’s disease	
Aminoacyl-tRNA biosynthesis	
Amino sugar and nucleotide sugar metabolism	
Amyotrophic lateral sclerosis (ALS)	
Androgen and estrogen metabolism	
Antigen processing and presentation	
Apoptosis	
Arachidonic acid metabolism	
Arginine and proline metabolism	
Ascorbate and aldarate metabolism	
Autoimmune thyroid disease	
Axon guidance	
Basal cell carcinoma	
Basal transcription factors	
Base excision repair	
B cell receptor signaling pathway	
beta-Alanine metabolism	
Biosynthesis of unsaturated fatty acids	
Bladder cancer	
Butanoate metabolism	
Caffeine metabolism	
Calcium signaling pathway	
Cell adhesion molecules (CAMs)	
Cell cycle	
Chemokine signaling pathway	
Chondroitin sulfate biosynthesis	
Chronic myeloid leukemia	
Citrate cycle (TCA cycle)	
Colorectal cancer	
Complement and coagulation cascades	
Cyanoamino acid metabolism	
Cysteine and methionine metabolism	
Cytokine-cytokine receptor interaction	
D-Arginine and D-ornithine metabolism	
D-Glutamine and D-glutamate metabolism	
Dilated cardiomyopathy	
DNA replication	
Dorso-ventral axis formation	
Drug metabolism - cytochrome P450	
Drug metabolism - other enzymes	
ECM-receptor interaction	
Endocytosis	
Endometrial cancer	
Epithelial cell signaling in Helicobacter pylori infection	
ErbB signaling pathway	
Ether lipid metabolism	
Fatty acid biosynthesis	
Fatty acid elongation in mitochondria	
Fatty acid metabolism	
Fc epsilon RI signaling pathway	
Fc gamma R-mediated phagocytosis	
Focal adhesion	
Folate biosynthesis	
Fructose and mannose metabolism	
Galactose metabolism	
Gap junction	
Glioma	
Glutathione metabolism	
Glycerolipid metabolism	
Glycerophospholipid metabolism	
Glycine, serine and threonine metabolism	
Glycolysis / Gluconeogenesis	
Glycosaminoglycan degradation	
Glycosphingolipid biosynthesis - globo series	
Glycosphingolipid biosynthesis - lacto and neolacto series	
Glyoxylate and dicarboxylate metabolism	
GnRH signaling pathway	
Graft-versus-host disease	
Hedgehog signaling pathway	
Hematopoietic cell lineage	
Heparan sulfate biosynthesis	
Histidine metabolism	
Homologous recombination	
Huntington’s disease	
Hypertrophic cardiomyopathy (HCM)	
Inositol phosphate metabolism	
Insulin signaling pathway	
Jak-STAT signaling pathway	
Keratan sulfate biosynthesis	
Leukocyte transendothelial migration	
Limonene and pinene degradation	
Linoleic acid metabolism	
Long-term depression	
Long-term potentiation	
Lysine biosynthesis	
Lysine degradation	
Lysosome	
Maturity onset diabetes of the young	
Melanogenesis	
Melanoma	
Metabolism of xenobiotics by cytochrome P450	
Methane metabolism	
Mismatch repair	
mTOR signaling pathway	
Natural killer cell mediated cytotoxicity	
Neuroactive ligand-receptor interaction	
Neurotrophin signaling pathway	
N-Glycan biosynthesis	
Nicotinate and nicotinamide metabolism	
Nitrogen metabolism	
NOD-like receptor signaling pathway	
Non-homologous end-joining	
Non-small cell lung cancer	
Notch signaling pathway	
Nucleotide excision repair	
O-Glycan biosynthesis	
Olfactory transduction	
One carbon pool by folate	
Other glycan degradation	
p53 signaling pathway	
Pancreatic cancer	
Pantothenate and CoA biosynthesis	
Parkinson’s disease	
Pathogenic Escherichia coli infection	
Pentose and glucuronate interconversions	
Pentose phosphate pathway	
Phenylalanine metabolism	
Phenylalanine, tyrosine and tryptophan biosynthesis	
Phosphatidylinositol signaling system	
Porphyrin and chlorophyll metabolism	
PPAR signaling pathway	
Primary bile acid biosynthesis	
Primary immunodeficiency	
Prion diseases	
Progesterone-mediated oocyte maturation	
Propanoate metabolism	
Prostate cancer	
Pyrimidine metabolism	
Pyruvate metabolism	
Regulation of actin cytoskeleton	
Renal cell carcinoma	
Renin-angiotensin system	
Retinol metabolism	
Riboflavin metabolism	
Ribosome	
RIG-I-like receptor signaling pathway	
RNA degradation	
Selenoamino acid metabolism	
Small cell lung cancer	
Sphingolipid metabolism	
Spliceosome	
Starch and sucrose metabolism	
Steroid biosynthesis	
Sulfur metabolism	
Synthesis and degradation of ketone bodies	
Systemic lupus erythematosus	
Taste transduction	
Taurine and hypotaurine metabolism	
T cell receptor signaling pathway	
Terpenoid backbone biosynthesis	
TGF-beta signaling pathway	
Thiamine metabolism	
Thyroid cancer	
Tight junction	
Toll-like receptor signaling pathway	
Tryptophan metabolism	
Type I diabetes mellitus	
Type II diabetes mellitus	
Tyrosine metabolism	
Ubiquinone and other terpenoid-quinone biosynthesis	
Ubiquitin mediated proteolysis	
Valine, leucine and isoleucine biosynthesis	
Valine, leucine and isoleucine degradation	
Vascular smooth muscle contraction	
VEGF signaling pathway	
Vibrio cholerae infection	
Viral myocarditis	
Vitamin B6 metabolism	
Wnt signaling pathway	
