# Supplementary material for: A Structure-Based Approach for Mapping Adverse Drug Reactions to the Perturbation of Underlying Biological Pathways
Source: PLoS One. 2010 Aug 23;5(8):e12063. doi: 10.1371/journal.pone.0012063 (PMC2925884; doi:10.1371/journal.pone.0012063)
Supplement: Table S5 — 830 protein structures used in this work. (0.28 MB RTF) [file pone.0012063.s005.rtf]

 PDB IDs	
   1a6q	1bhg	1bi8	1bj4	1blx	1bo1	1bui	1c25	
1c9h	1cm8	1cyn	1d1t	1d5r	1dig	1dpt	1e0a	
1eem	1efn	1ehw	1eqf	1eyb	1ezf	1f05	1fbv	
1fh0	1fjd	1fq1	1fsu	1fuj	1fw1	1fyj	1g55	
1gua	1gum	1hdr	1he5	1hlg	1htr	1hy3	1hzd	
1i10	1i3n	1i7k	1iau	1ik9	1ism	1itu	1ivh	
1jcn	1jd0	1jm4	1jmy	1jr2	1jvg	1k62	1k6m	
1kbh	1kr2	1kr5	1kt0	1l8k	1le7	1lt8	1m27	
1m3g	1m54	1m6b	1m6d	1mj4	1mkp	1mlw	1mq0	
1mzw	1nb5	1nfb	1nn5	1nnl	1nrg	1nst	1nuh	
1nuu	1nw3	1nzi	1o6x	1ohe	1orf	1oth	1p5j	
1p9o	1pbk	1pl0	1plo	1pq3	1pv8	1pyo	1q11	
1q22	1q3a	1q7l	1q7s	1q9s	1qha	1qip	1qk1	
1qr6	1qrk	1qvj	1qzu	1r03	1r8u	1r9o	1rfn	
1rhf	1rja	1rjb	1rkb	1rlw	1rqd	1rqq	1rxt	
1s5o	1s70	1sd2	1sg4	1so0	1spj	1t0l	1t2a	
1t2f	1t32	1t8u	1taz	1tdh	1tev	1tg6	1tjc	
1tpn	1tuz	1tzs	1u2h	1u31	1u3t	1uf0	1ufw	
1umk	1unl	1uw0	1uwy	1v04	1v3a	1v84	1v9v	
1vhr	1vjj	1vzo	1w2f	1w6k	1w6v	1wh0	1wl5	
1wlj	1wmh	1woj	1wrm	1wsv	1wvo	1wwc	1wwt	
1wxm	1wzw	1x0x	1x59	1x5d	1x5l	1x6a	1x6g	
1x9d	1xd3	1xdm	1xfb	1xm2	1xr0	1xrj	1xsc	
1xwn	1y97	1yb1	1yb5	1yh2	1yjx	1yrv	1yvh	
1yvj	1yxm	1yz4	1z6z	1z8d	1z8g	1zbq	1zd5	
1zd8	1zdn	1zjk	1zkc	1zn9	1znc	1zrh	1zrz	
1zs6	1zs9	1zsv	1zuo	1zx0	1zxn	2a14	2a1a	
2a1t	2a1x	2a2d	2a2n	2a5g	2a7l	2a7v	2a8b	
2a98	2acx	2aex	2ag5	2akz	2any	2ary	2aw5	
2awf	2ax4	2azt	2b25	2b3y	2b49	2b69	2bbw	
2bf8	2bgr	2bhl	2bug	2buj	2bwg	2bwj	2bzl	
2c05	2c11	2c2n	2c3t	2c46	2c4p	2c7s	2c95	
2c9y	2cb5	2ch6	2cjz	2cke	2clq	2coa	2coe	
2coj	2coo	2cq8	2cw6	2cyx	2d06	2d0j	2d7r	
2d9z	2daq	2ddy	2dfd	2djg	2djv	2dk6	2dlh	
2dm0	2dw5	2dyl	2dyn	2e0t	2e1q	2e2w	2e5g	
2edy	2ejm	2eli	2esb	2esl	2etl	2eva	2exe	
2f1o	2f29	2f2c	2f3m	2f4w	2f57	2f6q	2f8a	
2f9p	2f9q	2ffv	2fgb	2fh7	2fk9	2fue	2fun	
2fv7	2fvz	2fw2	2fy5	2g45	2g6z	2g76	2gdz	
2gjt	2gk9	2gl6	2gmi	2gmv	2gtr	2gu8	2gv7	
2gvj	2gwf	2h11	2h2u	2h31	2h5g	2h63	2h7v	
2hak	2hda	2he3	2hfy	2hgs	2hhf	2hhj	2hhm	
2hi4	2hq6	2hrb	2hrr	2hsp	2htf	2hve	2hvx	
2hw5	2hw6	2hw7	2hxp	2i0e	2i3y	2i50	2i5x	
2i6b	2i6l	2i7n	2i7p	2i9p	2ibi	2ibn	2iby	
2ic1	2ick	2idx	2iik	2iip	2img	2ipj	2iq1	
2ivv	2iwi	2iwz	2izu	2izz	2j0f	2j0t	2j3n	
2j4e	2j7t	2j9f	2jaj	2jam	2jbm	2jc6	2jcm	
2jev	2jii	2jis	2jlp	2jwx	2k4p	2k7l	2k8v	
2ka6	2nn7	2nq7	2nqa	2nsm	2nt2	2nz2	2nz6	
2nz8	2nzt	2o0l	2o2k	2o2v	2o36	2o3h	2o6l	
2oat	2ob4	2obi	2obv	2ocp	2oid	2ok3	2ok5	
2okj	2okk	2okn	2oo0	2ooq	2opb	2orv	2ou2	
2oud	2ow2	2ozo	2p02	2p0w	2p31	2p69	2p8e	
2p8u	2pa5	2pbc	2pbn	2pd6	2pe4	2pfr	2pid	
2pla	2pn8	2pny	2pph	2ppl	2pq5	2pq8	2pqf	
2pqt	2pvs	2px6	2pxx	2pzd	2pzg	2q0n	2q3z	
2q4r	2q4v	2q50	2q51	2q5a	2q5e	2q5i	2q71	
2q8h	2q8i	2q9g	2q9p	2qb5	2qbx	2qc8	2qd5	
2qep	2qg4	2qgx	2qjb	2qjf	2qnk	2qp4	2qpj	
2qpw	2qq2	2qrv	2qt1	2qtz	2quk	2qv2	2qy0	
2qyn	2r0n	2r17	2r2p	2r37	2r3a	2r3v	2r5t	
2r6k	2r99	2r9o	2r9p	2ra3	2rc4	2rda	2rdw	
2red	2rf0	2rf5	2rfe	2rgz	2riq	2rj9	2rmk	
2rop	2src	2uui	2uv2	2uvq	2uw2	2uzg	2uzp	
2uzq	2v1y	2v3s	2v4w	2v62	2v7o	2v9j	2v9y	
2vag	2vcy	2vd6	2vfa	2vgi	2vh7	2vhf	2vig	
2vki	2vkq	2vkt	2vl9	2vle	2vn0	2vo1	2vom	
2vph	2vr2	2vsw	2vt5	2vux	2vx1	2vx3	2vxo	
2vz2	2w0j	2w2x	2w4l	2w4m	2w4o	2w5h	2w73	
2wef	2wgh	2yr3	2ys0	2ys1	2ys2	2ysx	2yu2	
2yu7	2yuk	2yuq	2yuu	2yvq	2yz8	2z0p	2z4f	
2z5d	2z5y	2z6w	2z7s	2zb8	2zep	2zj4	2zmf	
2znv	2zoq	2zox	3b2z	3b3l	3b6h	3b7k	3b7o	
3b7x	3bbf	3bc3	3bce	3bd9	3bea	3beg	3bg8	
3bg9	3bhd	3bhm	3bic	3biy	3bj5	3bjc	3bju	
3bkl	3blr	3bm4	3bpt	3bq7	3bqr	3brb	3brt	
3bsq	3bsu	3bt1	3bt2	3bwy	3bz3	3bzh	3c0i	
3c10	3c3c	3c3t	3c4f	3c5g	3c6m	3c7x	3cbp	
3cd3	3cdb	3chs	3cik	3cjk	3ckh	3cki	3ckk	
3ckx	3cmq	3coi	3cok	3com	3cos	3cp6	3cpu	
3cr0	3crl	3cry	3ctr	3ctt	3ctz	3cu0	3cuk	
3cxs	3cyi	3cyn	3czd	3czy	3d2r	3d3l	3d3p	
3d44	3d4j	3d4q	3d5e	3d68	3d7c	3d7h	3d91	
3da2	3dai	3dak	3dar	3dax	3dc1	3dcy	3ddu	
3ddw	3dkk	3dl9	3dlj	3dlm	3dls	3dlx	3dpf	
3dre	3dtc	3dtw	3dw8	3dwb	3dyd	3dys	3e04	
3e0j	3e0l	3e37	3e4c	3e5a	3e6i	3e77	3e7e	
3e7o	3e9k	3eab	3ebs	3ecn	3edh	3edr	3ee2	
3ee5	3ee6	3efh	3eig	3elb	3elp	3ene	3eo3	
3epb	3epp	3epz	3eq1	3eqi	3eqr	3erb	3es3	
3eu0	3euf	3evs	3exi	3eyh	3f0r	3f1a	3f2a	
3f2k	3f5u	3f6q	3f6y	3f80	3f82	3f8g	3f8u	
3f92	3f9n	3f9z	3fcx	3fe3	3fe4	3feg	3fhb	
3fhy	3fju	3fkt	3fl7	3flg	3fmd	3fme	3fqs	
3fup	3fvx	3fvy	3fxv	3fy0	3fy1	3fy2	3g15	
3g2f	3g33	3g3m	3g3n	3g5p	3gam	3geb	3gey	
3ggf	3ggs	3ghu	3gjt	3glk	3gm1	3goi	3goy	
3gp0	3gpc	3gro	3gtu	3gv4	3gxm	3gxt	3gzd	
3h2x	3h30	3h3c	3h6l	3h6o	3h8m	3h8q	3h9e	
3h9f	3h9r	3had	3hcu	3hmm	3lck	3lhm	3nos	
3pbh	3pdz	3usn	4fap	4nos	5grt	5hck	5hpg	
5pnt	6fit	6pah	9ca2	9gss	9jdw			
