# Supplementary material for: A Structure-Based Approach for Mapping Adverse Drug Reactions to the Perturbation of Underlying Biological Pathways
Source: PLoS One. 2010 Aug 23;5(8):e12063. doi: 10.1371/journal.pone.0012063 (PMC2925884; doi:10.1371/journal.pone.0012063)
Supplement: Table S6 — 506 ADRs used in this work. (0.23 MB RTF) [file pone.0012063.s006.rtf]

 ADRs	
abnormal electroencephalogram	
abnormal pigmentation	
acute bronchitis	
acute leukemia	
acute pancreatitis	
adenitis	
adenocarcinoma	
adenoma	
adrenal insufficiency	
alkalosis	
allergic alveolitis	
allergic rhinitis	
aml	
amylase increased	
amyloidosis	
anaphylactoid purpura	
aneurysm	
ankle edema	
anosmia	
aortic stenosis	
aphasia	
aphonia	
appendicitis	
arachnoiditis	
ards	
arterial insufficiency	
arteriosclerosis	
ascites	
aseptic meningitis	
aseptic necrosis	
aspiration pneumonia	
atelectasis	
atopic dermatitis	
atrial flutter	
atrophic vaginitis	
avascular necrosis	
av block first degree	
azotemia	
bacterial vaginosis	
balanitis	
basal cell carcinoma	
benign prostatic hyperplasia	
bigeminy	
bipolar disorder	
bladder carcinoma	
bleeding diathesis	
blepharitis	
blepharoconjunctivitis	
blepharoptosis	
blepharospasm	
blood dyscrasia	
breast cancer	
breast enlargement	
breast lump	
breast neoplasm	
breast swelling	
bronchiolitis	
bruxism	
bundle branch block	
burning sensation	
bursitis	
cachexia	
carcinoma	
cardiac murmur	
cardiac tamponade	
cardiomegaly	
cardiomyopathy	
carpal tunnel syndrome	
cerebral hemorrhage	
cerebral infarction	
cerebral ischemia	
cerebral thrombosis	
cerebrovascular disorder	
cervical cancer	
cervical erosion	
change in bowel habit	
cheilosis	
cholangitis	
cholecystitis	
cholelithiasis	
choreoathetosis	
chronic active hepatitis	
chronic lymphocytic leukemia	
chronic renal failure	
cirrhosis	
claudication	
coated tongue	
cold extremity	
collagen disorder	
colon cancer	
colour blindness	
complete heart block	
concentration impaired	
copd	
corneal opacity	
corneal ulcer	
coronary artery disease	
cryptococcosis	
cushing syndrome	
cutaneous candidiasis	
deep thrombophlebitis	
deep vein thrombosis	
dental abscess	
diabetic ketoacidosis	
diverticulitis	
duodenal ulcer	
duodenitis	
dysesthesia	
dyspareunia	
dysphonia	
dyspnea on exertion	
ear infection	
easy bruising	
ectropion	
electrolyte imbalance	
elevated liver enzyme	
elevated liver function test	
embolism	
emphysema	
encephalitis	
encephalopathy	
endocarditis	
endocrine disorder	
endometrial cancer	
endometriosis	
enteritis	
enterocolitis	
enuresis	
eosinophilic pneumonia	
epididymitis	
epidural abscess	
epigastric pain	
erythema nodosum	
esophageal spasm	
esophageal stricture	
esophageal ulcer	
esophageal varix	
esr increased	
exophthalmos	
eyelid disorder	
eye redness	
eye swelling	
facial paralysis	
fasciitis	
fat embolism	
fecal impaction	
fecal incontinence	
fibrocystic breast	
fibromyalgia syndrome	
fibrosing alveolitis	
fibrosis	
fistula	
folliculitis	
fungal dermatitis	
furunculosis	
galactorrhea	
gallbladder disease	
gangrene	
gastric carcinoma	
gastric stasis	
gastric ulcer	
gastritis hemorrhagic	
gastroenteritis viral	
glomerulonephritis	
glossodynia	
goiter	
granuloma	
gum hemorrhage	
hairy tongue	
halitosis	
hemangioma	
hematemesis	
hematochezia	
hemiparesis	
hemiplegia	
hemoptysis	
hemorrhoid	
hepatic coma	
hepatic encephalopathy	
hepatic necrosis	
hepatitis infectious	
hepatomegaly	
hepatorenal syndrome	
hernia	
herpes	
herpes zoster	
hiatal hernia	
hirsutism	
hordeolum	
hydrocephalus	
hydronephrosis	
hyperacusis	
hypercalcemia	
hypercarbia	
hyperchloremia	
hyperesthesia	
hyperhidrosis	
hyperkeratosis	
hypermagnesemia	
hypernatremia	
hyperparathyroidism	
hyperphosphatemia	
hyperpigmentation	
hyperprolactinemia	
hypersomnia	
hyperthyroidism	
hypertrichosis	
hypertriglyceridemia	
hypervitaminosis	
hypochloremia	
hypochromic anemia	
hypomagnesemia	
hypomenorrhea	
hypophosphatemia	
hypopigmentation	
hypothermia	
hypothyroidism	
hypoventilation	
ichthyosis	
idiopathic thrombocytopenic purpura	
impetigo	
infectious mononucleosis	
infertility	
inflammatory bowel disease	
inguinal hernia	
interstitial lung disease	
interstitial nephritis	
interstitial pneumonitis	
intestinal perforation	
intestinal ulcer	
intracranial hemorrhage	
iritis	
iron deficiency anemia	
irritable bowel syndrome	
ischemia	
ischemic colitis	
joint swelling	
keratitis	
keratoconjunctivitis	
keratoconjunctivitis sicca	
kidney pain	
labyrinthitis	
lacrimation disorder	
lactic dehydrogenase increased	
latent diabetes	
leukemia	
leukocytoclastic vasculitis	
leukoplakia of mouth	
lichen planus	
lipoma	
liver disease	
liver fatty	
lobar pneumonia	
lower respiratory infection	
lung cancer	
lymphangitis	
lymphatic disorder	
lymphedema	
lymphocytic leukemia	
lymphocytosis	
lymphoma	
lymphopenia	
macrocytosis	
macular degeneration	
malabsorption	
malformation	
malignant hyperthermia	
masculinization	
mastitis	
megacolon	
megaloblastic anemia	
melanoma	
melasma	
meningismus	
meningitis	
menometrorrhagia	
menorrhagia	
menstrual flow	
menstrual irregularity	
metabolic acidosis	
methemoglobinemia	
microcytic anemia	
miliaria	
mitral insufficiency	
movement disorder	
muscle atrophy	
myasthenia gravis	
myelodysplastic syndrome	
myelofibrosis	
myelogenous leukemia	
myocarditis	
myopia	
myositis	
nail disorder	
narrow angle glaucoma	
nasal polyp	
nasal septum perforation	
nephrogenic diabetes insipidus	
nephrolithiasis	
nephropathy	
nephrosis	
nephrotic syndrome	
nerve palsy	
neuralgia	
neurosis	
night blindness	
nipple discharge	
nodal tachycardia	
nodule	
ocular infection	
odynophagia	
onycholysis	
ophthalmoplegia	
optic atrophy	
optic neuritis	
orchitis	
osteoarthritis	
osteomalacia	
osteomyelitis	
osteonecrosis	
osteoporosis	
otitis	
otitis externa	
ovarian cancer	
ovarian cyst	
papilledema	
papilloma	
paralytic ileus	
paraparesis	
parkinson’s	
paronychia	
parotitis	
pathological fracture	
pelvic pain	
pericardial effusion	
pericarditis	
periodontal abscess	
periodontal disease	
periodontitis	
peripheral vascular disorder	
peritonitis	
personality disorder	
petit mal	
pheochromocytoma	
photopsia	
pigmentary retinopathy	
pleurisy	
pleuritic pain	
pms	
pneumocystis carinii pneumonia	
pneumothorax	
polyarteritis nodosa	
polycythemia	
polydipsia	
polymyalgia rheumatica	
polymyositis	
polyneuropathy	
polyp	
porphyria	
porphyria cutanea tarda	
positive ana	
postmenopausal bleeding	
postnasal drip	
priapism	
proctitis	
prostate cancer	
prostatic hypertrophy	
prostatic specific antigen increase	
prostatitis	
pruritus ani	
pseudomembranous colitis	
pseudotumor cerebri	
psoriasis	
psychomotor retardation	
ptosis	
pulmonary fibrosis	
pulmonary hypertension	
pulmonary infiltration	
puncture	
pure red cell aplasia	
pyelonephritis	
pyoderma	
pyuria	
radiculopathy	
reflux esophagitis	
refraction disorder	
regurgitation	
renal colic	
renal cyst	
renal insufficiency	
renal papillary necrosis	
renal tubular acidosis	
renal tumor	
respiratory alkalosis	
respiratory arrest	
respiratory failure	
restless legs syndrome	
retinal artery occlusion	
retinal detachment	
retinal hemorrhage	
retinal vein thrombosis	
retinitis	
retinopathy	
retroperitoneal fibrosis	
rhabdomyolysis	
rheumatism	
rheumatoid arthritis	
right heart failure	
right upper quadrant pain	
rosacea	
salivary gland enlargement	
salpingitis	
sarcoma	
sciatica	
scleritis	
scleroderma	
seasonal allergic rhinitis	
seborrhea	
sensory disturbance	
septicemia	
septic shock	
serotonin syndrome	
serum sickness	
siadh	
sialadenitis	
sick sinus syndrome	
sinus arrest	
sinus bradycardia	
sinus congestion	
sinus headache	
sinus tachycardia	
skin atrophy	
skin benign neoplasm	
skin carcinoma	
skin fissure	
skin irritation	
skin lesion	
skin nodule	
sleep apnea	
small intestinal obstruction	
somnambulism	
spinal cord disease	
splenic infarction	
splenomegaly	
spontaneous abortion	
status epilepticus	
sterility	
strabismus	
streptococcal pharyngitis	
stria	
stridor	
subarachnoid hemorrhage	
subdural hematoma	
supraventricular extrasystole	
supraventricular tachycardia	
synovitis	
tardive dyskinesia	
telangiectasia	
tendinitis	
tenesmus	
tenosynovitis	
testicle pain	
testicular atrophy	
testicular swelling	
tetany	
thrombophlebitis superficial	
thrombotic thrombocytopenic purpura	
thyroid disorder	
thyroiditis	
thyroid neoplasia	
tonsillitis	
torticollis	
tracheitis	
transient ischemic attack	
transverse myelitis	
tuberculosis	
type 2 diabetes	
ulcerative colitis	
unsteady gait	
uremia	
ureteral obstruction	
urethritis	
urinary hesitancy	
urinary urgency	
uterine fibroid	
uterine hemorrhage	
uveitis	
vaginal discharge	
vaginal hemorrhage	
vaginal pruritus	
valvular heart disease	
varicella	
varicose vein	
vascular anomaly	
vascular headache	
vasovagal reaction	
venous thrombosis	
verruca	
vestibular disorder	
vitamin b12 deficiency	
vitamin d deficiency	
vitreous detachment	
vitreous floater	
vulvovaginitis	
white blood cell count increased	
wound dehiscence	
