# Supplementary material for: A Structure-Based Approach for Mapping Adverse Drug Reactions to the Perturbation of Underlying Biological Pathways
Source: PLoS One. 2010 Aug 23;5(8):e12063. doi: 10.1371/journal.pone.0012063 (PMC2925884; doi:10.1371/journal.pone.0012063)
Supplement: Table S7 — 730 drugs used in this work. (0.25 MB RTF) [file pone.0012063.s007.rtf]

 PubChem compund ID (CID)	
   85	119	137	159	187	191	206	232	
247	298	401	444	450	453	564	581	
596	598	681	727	738	807	815	838	
853	861	937	942	951	1046	1065	1071	
1125	1134	1148	1206	1302	1546	1690	1775	
1935	1971	1986	2021	2022	2082	2083	2088	
2092	2099	2118	2123	2130	2133	2140	2141	
2145	2148	2153	2160	2162	2170	2171	2173	
2179	2182	2187	2215	2216	2232	2244	2249	
2265	2266	2267	2269	2274	2284	2308	2311	
2315	2344	2349	2366	2370	2375	2381	2431	
2435	2441	2443	2462	2471	2474	2476	2477	
2478	2484	2487	2519	2522	2524	2541	2550	
2551	2554	2559	2564	2575	2576	2578	2583	
2585	2609	2610	2617	2622	2631	2637	2646	
2650	2654	2655	2656	2658	2662	2666	2673	
2675	2678	2708	2712	2719	2720	2725	2726	
2727	2732	2733	2749	2751	2756	2762	2764	
2769	2771	2781	2786	2794	2800	2801	2802	
2803	2806	2812	2818	2895	2905	2907	2913	
2949	2951	2955	2958	2978	2983	2995	3000	
3003	3007	3009	3015	3016	3019	3032	3040	
3042	3043	3059	3062	3066	3075	3080	3100	
3105	3114	3117	3121	3125	3148	3151	3152	
3154	3156	3157	3158	3161	3168	3198	3203	
3219	3226	3241	3249	3251	3255	3261	3278	
3279	3285	3291	3292	3305	3308	3310	3324	
3325	3333	3339	3340	3342	3345	3348	3350	
3354	3355	3365	3366	3367	3372	3373	3375	
3379	3381	3382	3384	3385	3386	3387	3392	
3393	3394	3397	3403	3404	3405	3410	3414	
3417	3440	3446	3449	3454	3461	3463	3467	
3475	3478	3494	3510	3512	3518	3519	3553	
3559	3562	3598	3636	3637	3639	3640	3647	
3648	3652	3658	3661	3672	3675	3676	3685	
3687	3690	3696	3698	3702	3715	3737	3746	
3749	3750	3759	3763	3767	3779	3780	3783	
3784	3821	3823	3825	3826	3827	3869	3877	
3878	3883	3899	3902	3914	3915	3928	3929	
3948	3950	3954	3956	3957	3958	3961	3962	
3964	3998	4011	4030	4032	4033	4034	4036	
4043	4044	4046	4052	4053	4054	4057	4058	
4060	4062	4064	4075	4078	4086	4091	4095	
4100	4101	4107	4112	4114	4121	4138	4140	
4158	4159	4160	4163	4168	4170	4171	4173	
4174	4178	4189	4192	4195	4196	4197	4200	
4201	4205	4211	4236	4253	4259	4409	4411	
4419	4421	4425	4428	4436	4440	4449	4463	
4485	4493	4497	4506	4509	4510	4513	4536	
4539	4542	4543	4583	4585	4594	4595	4601	
4603	4607	4614	4616	4623	4634	4635	4638	
4645	4674	4675	4679	4689	4691	4723	4724	
4725	4727	4730	4736	4737	4739	4740	4745	
4748	4768	4771	4775	4819	4828	4829	4834	
4845	4856	4865	4870	4885	4891	4893	4894	
4900	4908	4909	4911	4913	4914	4915	4917	
4919	4920	4927	4934	4935	4943	4946	4976	
4991	4993	5002	5029	5035	5039	5052	5064	
5070	5071	5073	5077	5078	5090	5095	5155	
5193	5195	5203	5206	5210	5212	5215	5245	
5253	5267	5291	5297	5300	5318	5320	5333	
5342	5344	5352	5358	5359	5376	5379	5381	
5391	5394	5396	5401	5402	5403	5404	5408	
5412	5419	5426	5430	5452	5453	5454	5466	
5472	5478	5479	5487	5496	5503	5504	5505	
5508	5512	5514	5515	5516	5523	5526	5530	
5533	5538	5544	5546	5556	5566	5572	5578	
5582	5584	5591	5593	5596	5625	5645	5647	
5656	5665	5672	5718	5719	5721	5726	5731	
5732	5734	5735	5746	5775	5878	6256	6476	
6503	6691	7029	7187	7638	8612	9034	9433	
9904	10100	10548	10631	12536	12555	16231	16362	
16850	18140	19090	20585	23897	25419	27400	27661	
27686	30623	31378	31477	32170	32797	34312	34633	
36339	36811	39042	39507	39765	39860	40159	40704	
40976	41317	41693	41774	41781	42113	44564	47472	
47641	48041	48175	50294	50614	51263	51577	51634	
52421	54454	54547	54688	54786	54841	56339	56959	
57469	57537	59708	59768	60164	60184	60198	60612	
60613	60696	60707	60795	60815	60831	60835	60843	
60852	60854	60865	60877	60879	60937	60953	62867	
62924	62959	64147	65863	65999	68740	68844	71158	
71273	71301	71616	72054	72938	74989	77992	77993	
77999	82146	83786	87177	96312	104758	110634	110635	
115237	119182	119607	122316	123606	123620	123631	124087	
125017	125889	148211	150310	150311	150610	151165	152945	
153941	154059	156419	157922	170361	176870	197712	216239	
216326	222786	315411	443871	444033	504578	508230	657298	
667490	1349907	2761171	3062316	3085017	3086258	4630253	4659568	
4659569	5281007	5281104	5282044	5311048	5311181	5311297	5329102	
5353894	5353980	5362070	5462337	5473385	5487301	5493381	6398970	
6436173	9571074							
