# Supplementary material for: A Structure-Based Approach for Mapping Adverse Drug Reactions to the Perturbation of Underlying Biological Pathways
Source: PLoS One. 2010 Aug 23;5(8):e12063. doi: 10.1371/journal.pone.0012063 (PMC2925884; doi:10.1371/journal.pone.0012063)
Supplement: Table S9 — The 32 associations supported by the literature with the corresponding drug names. (Top) The 22 associations with stronger support. (Bottom) The 10 associations with moderate support. (0.06 MB RTF) [file pone.0012063.s009.rtf]

 ADRs	Pathways	Drugs	
   Cerebral infarction	Alzheimer’s disease	ziprasidone, memantine, phenytoin, lenalidomide, lansoprazole, cefpodoxime, fosphenytoin, BCNU, tizanidine, irinotecan, pregabalin, celecoxib, pramipexole, formoterol, anastrozole, moxifloxacin	
Osteoporosis	Type II diabetes mellitus	tamoxifen, ciclesonide, tramadol, prednisolone, letrozole, mycophenolic acid, brimonidine, budesonide, fluticasone, lenalidomide, cortisol, topiramate, methotrexate, valdecoxib, pregabalin, zolmitriptan, omeprazole, prednisone, pramipexole, fludrocortisone, vitamin D, cyproterone, dexamethasone, cortisone, methylprednisolone, exemestane	
Lymphoma	Retinol metabolism	daunorubicin, phenytoin, lenalidomide, ropinirole, hydromorphone, imiquimod, cidofovir, valdecoxib, zoledronic acid, mephenytoin, clomipramine, lamivudine, melphalan, ethotoin, dantrolene, riluzole, amsacrine, mycophenolic acid, didanosine, foscarnet, irinotecan, pregabalin, nitrogen mustard, selegiline, pramipexole	
Hernia	Prostate cancer	rabeprazole, clopidogrel, methylphenidate, diclofenac, imiquimod, fenofibrate, omeprazole, sildenafil, bupivacaine, Ras, doxazosin, thalidomide, tramadol, nicotinic acid, mycophenolic acid, pantoprazole, tolcapone, bicalutamide, pregabalin, indapamide, gliclazide, formoterol, moxifloxacin, cyproterone	
Parkinson’s	Pyruvate metabolism	methyldopa, olanzapine, memantine, reserpine, aripiprazole, lamotrigine, 9-hydroxyrisperidone, alpha-methyl-p-tyrosine, indomethacin, 5-fluorouracil, fentanyl, valproic acid, medroxyprogesterone, trifluoperazine, thiothixene, risperidone, sumatriptan, buspirone, THA, cisapride, chlorpromazine, fluoxetine, perphenazine, quetiapine, fluphenazine, molindone, thioridazine, loxapine, haloperidol, 5-fluorocytosine, prochlorperazine, clozapine, mesoridazine	
Breast cancer	Non-homologous end-joining	pemetrexed, zolmitriptan, progesterone, spironolactone, tramadol, paroxetine, bupropion, tegaserod, paricalcitol, naproxen, clomiphene citrate, pregabalin, selegiline, pergolide, pramipexole	
Pelvic pain	Cell cycle	tamoxifen, atomoxetine, rabeprazole, phenytoin, lenalidomide, fosphenytoin, aripiprazole, nifedipine, fentanyl, estradiol, meropenem, mycophenolic acid, testosterone, rofecoxib, doxorubicin, naproxen, bicalutamide, quetiapine, pregabalin, estrogen, topotecan, clindamycin, mifepristone, danazol, formoterol, aprepitant, moxifloxacin, clonazepam	
Fibrosis	Nicotinate and nicotinamide metabolism	meclofenamate, cisplatin, 2-chlorodeoxyadenosine, nitrofurantoin, mycophenolic acid, busulfan, mecamylamine, anagrelide, cyclophosphamide, D-penicillamine, tizanidine, gentamicin, methotrexate, cimetidine, fludarabine, 6-thioguanine, flurbiprofen, omeprazole, 5-aminosalicylic acid, CCNU, BCNU, capecitabine, 5-fluorouracil, mitomycin C, melphalan	
hepatic encephalopathy	Thiamine metabolism	flutamide, cyclophosphamide, amiloride, furosemide	
Melanoma	Hedgehog signaling pathway	azathioprine, rabeprazole, ropinirole, valdecoxib, medroxyprogesterone, mycophenolic acid, paroxetine, pregabalin, selegiline, pramipexole	
Prostatitis	Pathogenic Escherichia coli infection	atomoxetine, tramadol, paroxetine, venlafaxine, pramipexole, trovafloxacin	
Alkalosis	Type II diabetes mellitus	prednisolone, phenytoin, cortisol, fosphenytoin, prednisone, fludrocortisone, indomethacin, dexamethasone, triamcinolone, mycophenolic acid, metolazone, CLT, naproxen, cortisone, methylprednisolone	
Stria	Heparan sulfate biosynthesis	prednisolone, prednicarbate, amcinonide, fluticasone, cortisol, alclometasone, halobetasol propionate, apolar, fluocinolone acetonide, donepezil, fludrocortisone, halcinonide, desoximetasone, dexamethasone, triamcinolone, flurandrenolone, budesonide, flumethasone, clobetasol, venlafaxine, beclomethasone, fluocinonide, mometasone	
tuberculosis	Glycosaminoglycan degradation	delavirdine, norethisterone, mycophenolic acid, efavirenz	
herpes zoster	Glycosaminoglycan degradation	delavirdine, 2-chlorodeoxyadenosine, temozolomide, methotrexate, fenofibrate, valdecoxib, flurbiprofen, fentanyl, doxazosin, estradiol, tramadol, riluzole, nicotinic acid, mycophenolic acid, pantoprazole, rofecoxib, doxorubicin, tolcapone, naproxen, bicalutamide, pregabalin, nitrogen mustard, tiotropium, celecoxib, pramipexole, formoterol, capecitabine, DCF	
Cirrhosis	Nicotinate and nicotinamide metabolism	ads, torasemide, acamprosate, rabeprazole, simvastatin, fluvastatin, methotrexate, diclofenac, ICL670, chlorpromazine, tizanidine, mirtazapine, venlafaxine, cyproterone	
Ascites	Nicotinate and nicotinamide metabolism	retinoic acid, rabeprazole, amrinone, hydromorphone, fentanyl, valganciclovir, thalidomide, ofloxacin, mycophenolic acid, etoposide, cyclophosphamide, doxorubicin, irinotecan, Bax, pregabalin, 6-thioguanine, pramipexole, capecitabine, cyproterone	
Meningitis	Heparan sulfate biosynthesis	daunorubicin, phenytoin, piroxicam, sulfasalazine, diclofenac, fosphenytoin, flurbiprofen, BCNU, baclofen, ranitidine, ropivacaine, bupivacaine, thalidomide, lidocaine, mycophenolic acid, mepivacaine, clonidine, ketorolac, foscarnet, thiotepa, tolcapone, naproxen, etodolac, pregabalin, oxaprozin, carbamazepine, mefenamic acid	
Wound dehiscence	Glycosaminoglycan degradation	ertapenem, fluocinolone acetonide, baclofen, valganciclovir, aprepitant	
Amylase increased	Glycosaminoglycan degradation	delavirdine, atovaquone, linezolid, valproic acid, rivastigmine, SU11248, tramadol, tigecycline, bexarotene, cytosine arabinoside, Bax, pregabalin, moxifloxacin	
Fibrosis	Keratan sulfate biosynthesis	meclofenamate, 2-chlorodeoxyadenosine, mecamylamine, D-penicillamine, gentamicin, methotrexate, fludarabine, flurbiprofen, omeprazole, 5-aminosalicylic acid, BCNU, 5-fluorouracil, melphalan, cisplatin, nitrofurantoin, mycophenolic acid, busulfan, anagrelide, cyclophosphamide, tizanidine, cimetidine, 6-thioguanine, CCNU, capecitabine, mitomycin C	
Ptosis	Type II diabetes mellitus	fluorometholone, isosorbide-5-mononitrate, prednisolone, oxcarbazepine, ropinirole, cetirizine, guanethidine, fluocinolone acetonide, timolol, baclofen, remifentanil, ropivacaine, Ets, gatifloxacin, ketorolac, naproxen, pregabalin, HMS, loxapine, levobunolol, carteolol	
Aseptic meningitis	Systemic lupus erythematosus	trimethoprim, sulfasalazine, ibuprofen, methotrexate, ketoprofen, metronidazole, bupivacaine, sulindac, pentoxifylline	
Lymphoma	Heparan sulfate biosynthesis	daunorubicin, phenytoin, lenalidomide, ropinirole, hydromorphone, imiquimod, cidofovir, valdecoxib, zoledronic acid, mephenytoin, clomipramine, lamivudine, melphalan, ethotoin, dantrolene, riluzole, amsacrine, mycophenolic acid, didanosine, foscarnet, irinotecan, pregabalin, nitrogen mustard, selegiline, pramipexole	
Skin carcinoma	Lysosome	delta-aminolevulinic acid, rabeprazole, diclofenac, sildenafil, Ras, mycophenolic acid, bicalutamide, pregabalin, selegiline	
Alkalosis	Biosynthesis of unsaturated fatty acids	prednisolone, phenytoin, cortisol, fosphenytoin, prednisone, fludrocortisone, indomethacin, dexamethasone, triamcinolone, mycophenolic acid, metolazone, CLT, naproxen, cortisone, methylprednisolone	
Hyperparathyroidism	Autoimmune thyroid disease	tiludronate, valdecoxib, omeprazole, clodronate	
Fibrosis	Metabolism of xenobiotics by cytochrome P450	meclofenamate, cisplatin, 2-chlorodeoxyadenosine, nitrofurantoin, mycophenolic acid, busulfan, mecamylamine, anagrelide, cyclophosphamide, D-penicillamine, tizanidine, gentamicin, methotrexate, cimetidine, fludarabine, 6-thioguanine, flurbiprofen, omeprazole, 5-aminosalicylic acid, CCNU, BCNU, capecitabine, 5-fluorouracil, mitomycin C, melphalan	
Vitamin d deficiency	Autoimmune thyroid disease	tiludronate, nitrofurantoin, pramipexole	
Skin carcinoma	Androgen and estrogen metabolism	delta-aminolevulinic acid, rabeprazole, diclofenac, sildenafil, Ras, mycophenolic acid, bicalutamide, pregabalin, selegiline	
Pigmentary retinopathy	Sulfur metabolism	trifluoperazine, chlorpromazine, perphenazine, molindone, prochlorperazine	
Esr increased	Parkinson’s disease	retinoic acid, amrinone, tramadol, ciprofloxacin, budesonide, fluoxetine, pregabalin, selegiline, methysergide, moxifloxacin	
